# Improving infection control in developing countries: the infection control assessment tool

**DOI:** 10.1186/1753-6561-5-S6-O18

**Published:** 2011-06-29

**Authors:** C Huskins, D Ross-Degnan, DA Goldmann

**Affiliations:** 1 MAYO CLINIC, Rochester, Boston, USA; 2 Harvard Medical School, Boston, USA; 3 Harvard Pilgrim Health Care Institute, Boston, USA; 4 Institute for Healthcare Improvement, Boston, USA

## Introduction / objectives

The burden of healthcare-associated infections (HAI) is large in developing countries. There is an urgent need to improve HAI prevention in these countries.

## Methods

We developed the Infection Control Assessment Tool (ICAT) under the auspices of the Rational Pharmaceutical Management Program at Management Sciences for Health (Washington, DC) with support from the United States Agency for International Development. ICAT enables users with limited infection control expertise to complete hospital-wide, unit-based, or problem-based evaluations of existing infection control infrastructures and practices. ICAT includes 21 modules, each focused on a particular topic (e.g., hand hygiene, isolation and standard precautions, disinfection and sterilization) or department/ward (e.g., labor and delivery, intensive care, medical/surgical ward). ICAT provides a scoring system to evaluate the findings and makes practical, low-cost recommendations for improvement based on guidelines from international organizations (e.g., World Health Organization, Centers for Disease Control and Prevention) and experts.

## Results

With the assistance of hospital staff and governmental officials, ICAT was field-tested in acute care hospitals of different types in the Philippines and Uganda. ICAT was refined to ensure that commonly-encountered problems were identified clearly and that its recommendations for improvement were feasible in low resource settings.

## Conclusion

ICAT is a simple, practical tool to improve infection control in low-resource healthcare facilities. ICAT is ready for wider implementation to evaluate its effectiveness in reducing HAIs.

## Disclosure of interest

None declared.

